# Evolution of differences in clinical presentation across epidemic waves among patients with COVID-like-symptoms who received care at the Mexican Social Security Institute

**DOI:** 10.3389/fpubh.2023.1102498

**Published:** 2023-02-27

**Authors:** Gustavo Olaiz, Stefano M. Bertozzi, Arturo Juárez-Flores, Víctor H. Borja-Aburto, Félix Vicuña, Iván J. Ascencio-Montiel, Juan Pablo Gutiérrez

**Affiliations:** ^1^Center for Policy, Population and Health Research, School of Medicine, National Autonomous University of Mexico, Mexico City, Mexico; ^2^School of Public Health, University of California, Berkeley, Berkeley, CA, United States; ^3^Department of Global Health, University of Washington, Seattle, WA, United States; ^4^National Institute of Public Health, Mexico (INSP), Cuernavaca, Mexico; ^5^Education and Research Unit, Mexican Social Security Institute, Mexico City, Mexico; ^6^Coordination of Epidemiological Surveillance, Mexican Institute of Social Security, Mexico City, Mexico

**Keywords:** COVID-19, Mexico, symptoms and signs, social security, big data

## Abstract

**Background:**

Timely monitoring of SARS-CoV-2 variants is crucial to effectively managing both prevention and treatment efforts. In this paper, we aim to describe demographic and clinical patterns of individuals with COVID-19-like symptoms during the first three epidemic waves in Mexico to identify changes in those patterns that may reflect differences determined by virus variants.

**Methods:**

We conducted a descriptive analysis of a large database containing records for all individuals who sought care at the Mexican Social Security Institute (IMSS) due to COVID-19-like symptoms from March 2020 to October 2021 (4.48 million records). We described the clinical and demographic profile of individuals tested (3.38 million, 32% with PCR and 68% with rapid test) by test result (positives and negatives) and untested, and among those tested, and the changes in those profiles across the first three epidemic waves.

**Results:**

Individuals with COVID-19-like symptoms were older in the first wave and younger in the third one (the mean age for those positive was 46.6 in the first wave and 36.1 in the third wave; for negatives and not-tested, the mean age was 41 and 38.5 in the first wave and 34.3 and 33.5 in the third wave). As the pandemic progressed, an increasing number of individuals sought care for suspected COVID-19. The positivity rate decreased over time but remained well over the recommended 5%. The pattern of presenting symptoms changed over time, with some of those symptoms decreasing over time (dyspnea 40.6 to 14.0%, cough 80.4 to 76.2%, fever 77.5 to 65.2%, headache 80.3 to 78.5%), and some increasing (odynophagia 48.7 to 58.5%, rhinorrhea 28.6 to 47.5%, anosmia 11.8 to 23.2%, dysgeusia 11.2 to 23.2%).

**Conclusion:**

During epidemic surges, the general consensus was that any individual presenting with respiratory symptoms was a suspected COVID-19 case. However, symptoms and signs are dynamic, with clinical patterns changing not only with the evolution of the virus but also with demographic changes in the affected population. A better understanding of these changing patterns is needed to improve preparedness for future surges and pandemics.

## Introduction

Coronavirus disease (COVID-19) originated in Wuhan Province, China, in 2019 (1). It is caused by the SARS-CoV-2 virus which causes mild or severe clinical manifestations that principally affect the respiratory system. The COVID-19 pandemic has had repeated exacerbations as new variants emerge that are more infectious and/or able to evade existing immune defenses (2–7).

Like other RNA viruses, SARS-CoV-2 is prone to genetic evolution creating variants with characteristics that are different from those of their parent strains. Many SARS-CoV-2 variants have been described during this pandemic, but only a few are considered variants of concern because of significant changes in infectiousness, pathogenicity, immune escape, or resistance to treatments (8, 9).

Mexico has experienced at least four waves of COVID. The original strain of SARS-CoV-2 caused the first wave, the second was dominated by the Alpha variant, the third by Delta, and the fourth by Omicron (10, 11).

In Mexico, the first COVID-19 case was identified on February 27, 2020. By March 23, the federal government closed all schools and on March 30 declared a health emergency, suspending all non-essential activities and calling upon the population to stay at home, but without enforcing a lockdown. By June 1, suspended activities were allowed to restart in some areas and with restrictions in terms of capacity. By the end of 2020, most states had restarted activities, although still with some restrictions (12).

COVID-19 vaccination started in Mexico in December 2020 for health professionals. In February 2021 individuals 60 years and older became eligible, followed progressively by including younger individuals by decade. By October 31, 2021 (the period covered in our analyses), an estimated 58.5% of the population had received at least one dose of the vaccine; this increased to 77.7% by the end of 2022 (13). Available data on vaccination coverage indicates that by the second semester of 2021, 73.8% of all individuals 18 years and older received at least one dose of the COVID-19 vaccine, with an increasing proportion by age going from 51.3% among those 18–29 years, 76.8% among those 30–39 years, 82.5% for those 40–49 years, 85.4% for those 50–59 years, and 87.3% for those 60 years and older, with negligible differences by sex (14).

Monitoring the emergence of new SARS-CoV-2 variants is crucial to effectively managing prevention efforts (including vaccine recommendations) and treatment recommendations in light of emerging resistance to existing treatments (10, 11).

This study aims to describe and analyze the clinical and demographic characteristics of COVID-19 cases across the first three waves in patients treated at the Mexican Social Security Institute (IMSS).

## Methodology

This study is a cross-sectional analysis of the data recorded in the Epidemiological Surveillance Online Notification System (SINOLAVE) of the Mexican Social Security Institute (IMSS). We have previously described the data sources (15). SINOLAVE includes data of all people with symptoms suspicious of COVID-19 who were treated in IMSS health facilities, regardless of whether they were IMSS beneficiaries or not.

We included all cases from the SINOLAVE dataset from March 2020 through October 2021. The database included the most relevant symptoms and signs (16).

We used “suspect case” and “severe case” as defined by the Ministry of Health of Mexico. A suspect case is a person with at least one major sign or symptom (cough dyspnea, fever or headache) and at least one of the minor signs or symptoms (myalgias, arthralgias, odynophagia, chills, chest pain, rhinorrhea, polypnea, anosmia, dysgeusia, and conjunctivitis). A severe case is a suspect case who also presents with dyspnea or chest pain (17).

We considered three pandemic waves based on a visual examination of the pandemic curve: March-October 2020, November 2020 to March 2021, and April 2021-October 2021, inclusive.

### Data analysis

Individual observations were classified according to their SARS-CoV-2 status (untested, and tested positive/negative), as well as by epidemic wave. We then compared their demographic characteristics, as well as the prevalence of signs and symptoms. Because the demographic characteristics of people with COVID-like-symptoms vary across the waves, we standardized the prevalence of signs and symptoms using the age and sex distribution of wave 2. We also estimated the prevalence of signs and symptoms by age group and sex.

During the first wave most of the test results reported were from PCR assays; during waves 2 and 3 rapid tests were more common. For the analysis reported here we did not distinguish between the diagnostic test used, as this was the approach at IMSS for immediate clinical decisions based on the high sensitivity and specificity of the rapid tests (18, 19). If any test had a positive result, the person was assumed to be positive. Overall, 68% of cases were confirmed with rapid test, from almost none in the first wave, to 53% during the second wave and 89% during the third wave.

Given that the analysis used all recorded cases, we treated this group as a census of all individuals who sought care at IMSS and were considered suspect COVID cases. We tested the difference in the age and sex standardized proportions for each sign and symptom between wave 1 and 2, wave 1 and 3, and wave 2 and 3 using a z-test between each pair of waves' proportions.

This descriptive analysis was implemented in Stata software version 15.

## Results

We analyzed data from 4.48 million individuals who sought care at IMSS health facilities and were identified by healthcare providers as suspect COVID cases between March 1^st^, 2020, and November 30, 2021. Approximately 75% of the cases (3.38 million) were tested for SARS-CoV-2, either with PCR or a rapid test; the remaining 25% (1.10 million) were not tested (Table 1).

**Table 1:** Characteristics of suspect cases by wave and testing status.

	**Wave 1**			**Wave 2**			**Wave 3**		
	**Positive**	**Negative**	**Untested**	**Positive**	**Negative**	**Untested**	**Positive**	**Negative**	**Untested**
Observations	166,455	131,625	384,230	506,805	717,770	593,666	689,875	1,168,437	123,396
Male (%)	52.9%	44.4%	50.5%	50.9%	44.5%	48.2%	51.4%	44.1%	42.5%
Age (mean) (S.D.)	46.6 (16.3)	41.0 (16.7)	38.5 (14.7)	43.8 (16.8)	37.9 (16.5)	37.0 (14.7)	36.1 (15.6)	34.3 (16.4)	33.5 (15.4)

The percentage of patients with COVID-like-symptoms who were tested increased over time: during the first wave 43.7% of suspected cases were tested for SARS-CoV-2, with 67.3 and 93.8% tested in waves 2 and 3, respectively. Including all waves, 40.3% of the tests (1.36 million individuals) tested positive for SARS-CoV-2 (Table 1).

The positivity rate decreased with the increase in the proportion of suspected cases tested: 55.8% were positive during the first wave, 41.4% during the second, and 37.1% during the third (Table 1).

### Demographic profile

With respect to the demographic profile of suspect COVID-19 cases, men were overrepresented among those who tested positive while women were overrepresented among those who did not test or who tested negative. During the first wave, men comprised s 52.9, 44.4, and 50.5% of positive cases, negative cases and untested cases, respectively; 50.9, 44.5, and 48.2% during the second wave, and 51.4, 44.1, and 42.5% for the third (Table 1).

Average age decreased over time for the three groups (positive, negative, and untested). Among COVID-positive cases, there was a decrease of ten years in the average age: 46.6 years in the first wave, 43.8 years in the second, and 36.1 years in the third. Average age also decreased among those who tested negative–although to a lesser degree; 41.0 years in wave 1, 37.9 years in wave 2, and 34.3 years in wave 3. Among the untested, the average age was 38.5 years in wave 1, 37.0 years in wave 2, and 33.5 years in wave 3. The average age of positive cases was consistently higher than the other two categories across all three waves (Table 1).

Table 2 shows the change in age distribution through the three epidemic waves by SARS-CoV-2 test status reporting the age- and sex-specific rates per 100 thousand individuals in Mexico. For those with a positive SARS-CoV-2 test, rates were higher for older individuals among males in the first and second waves and then for 20 to 39 in the third wave. For females, higher rates were for those 70 to 79 and 40 to 49 in the first wave, then those 30 to 49 years in the second wave, and finally, those 20 to 39 for the third wave. It is relevant to highlight that while the increase in the rate per 100 thousand individuals from wave 1 to wave 3 for older individuals was about 1.5-fold, it was about 10-fold for those 20 to 39 years and 20-fold for those 0 to 19 years. Age patterns did not change as much for those negative and non-tested.

**Table 2:** Age- and sex-specific rates per 100 thousand individuals of positive, negative and non-tested patients with COVID-like-symptoms.

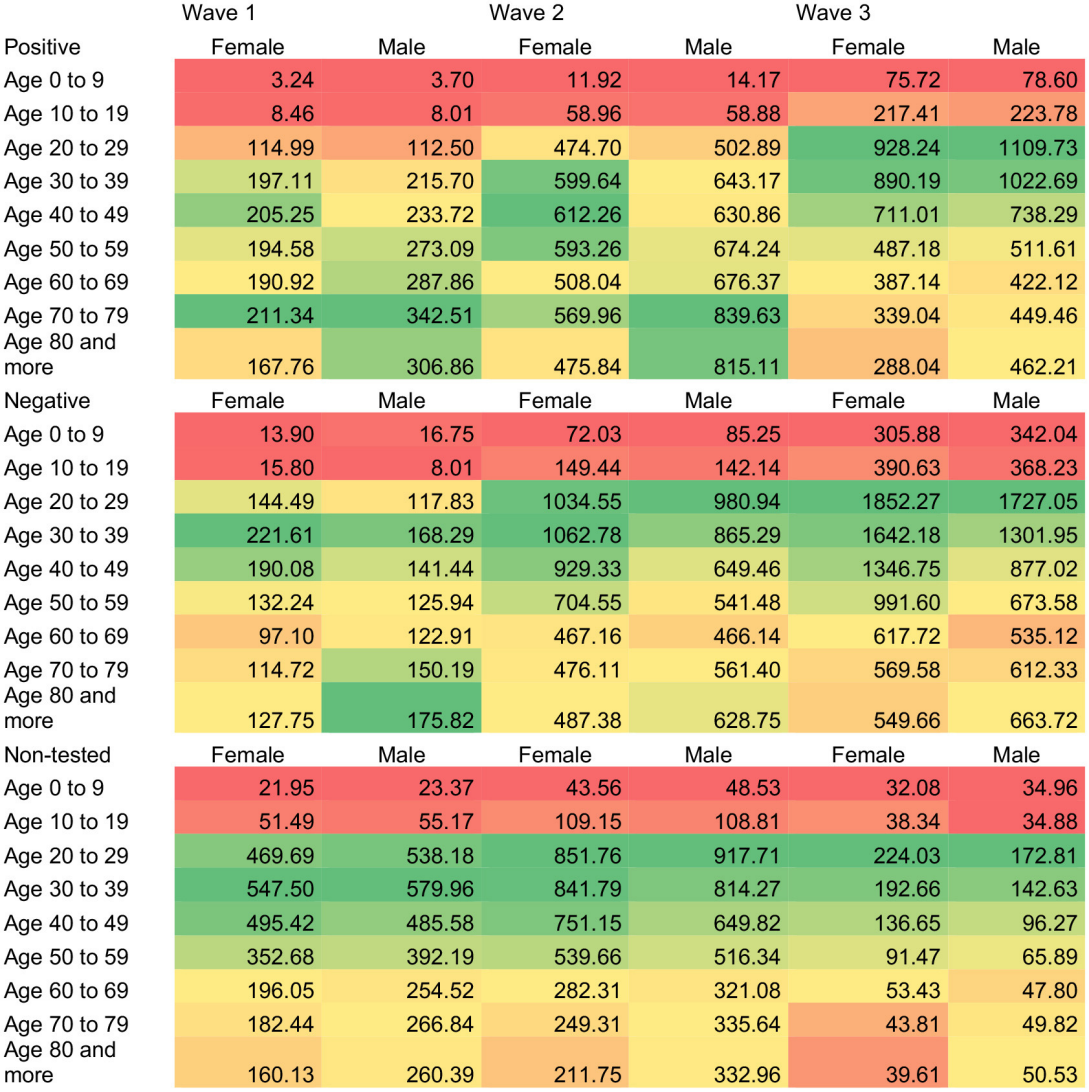

The color scale indicates lower (red) to higher (green) rates for each wave and sex by age-groups.

### Signs and symptoms

Table 3 reports the standardized prevalence of signs and symptoms among all suspect cases by testing status and epidemic wave. Despite the changes over time described above in the characteristics of the population becoming infected and the changes in the virus from wave to wave as new variants become dominant, the five most common signs and symptoms (headache, cough, fever, myalgia, and arthralgia) have not changed. This is true not only among confirmed positive cases but also among negatives and untested suspected cases.

**Table 3:** Prevalence of symptoms among suspect cases by wave and testing status.

	**Wave 1**			**Wave 2**			**Wave 3**		
**Symptom**	**Positive**	**Negative**	**Untested**	**Positive**	**Negative**	**Untested**	**Positive**	**Negative**	**Untested**
Cough	79.9%	68.1%	49.3%	75.0%	62.0%	35.5%	77.0%	63.1%	43.4%
Headache	81.0%	79.3%	54.1%	77.6%	75.2%	39.5%	77.4%	75.9%	47.7%
Fever	77.2%	63.3%	48.1%	61.9%	47.8%	28.7%	64.1%	47.4%	35.6%
Myalgia	66.0%	56.9%	41.8%	61.6%	52.2%	29.2%	59.3%	50.5%	34.8%
Arthralgia	60.5%	51.2%	37.5%	55.3%	45.1%	25.6%	52.5%	42.5%	30.2%
Generalized malaise	53.4%	46.3%	29.5%	43.8%	33.7%	18.6%	35.9%	30.5%	20.0%
Irritability (<5 years old)	0.3%	1.2%	0.6%	0.2%	0.9%	0.3%	0.2%	0.8%	0.3%
Odynophagia	49.4%	47.8%	34.4%	53.1%	54.2%	27.4%	57.0%	57.2%	35.1%
Dyspnea	37.5%	23.5%	14.2%	29.0%	14.4%	9.6%	19.0%	10.1%	7.9%
Shaking chills	40.1%	34.3%	22.5%	39.5%	31.7%	16.3%	31.2%	27.0%	17.1%
Thoracic pain	30.7%	24.8%	15.8%	24.7%	17.8%	9.9%	17.0%	13.7%	9.6%
Rhinorrhea	29.5%	28.3%	20.9%	38.1%	41.0%	20.6%	46.0%	44.4%	27.7%
Diarrhea	22.0%	23.1%	13.4%	14.5%	16.3%	7.1%	11.6%	15.2%	9.0%
Abdominal pain	13.9%	15.5%	7.6%	9.9%	10.9%	4.2%	7.9%	10.1%	5.2%
Anosmia	12.3%	6.0%	6.9%	25.6%	11.5%	10.5%	22.5%	8.4%	10.7%
Dysgeusia	11.6%	6.0%	6.8%	23.7%	11.2%	9.8%	20.7%	8.0%	10.0%
Conjunctivitis	8.4%	9.0%	5.1%	7.9%	7.7%	3.4%	7.2%	7.1%	3.8%
Prostration	5.5%	4.2%	2.4%	5.5%	2.9%	1.9%	3.6%	2.3%	1.6%
Cyanosis	3.3%	2.1%	1.0%	2.6%	1.0%	0.6%	1.2%	0.6%	0.5%
Polypnea	3.3%	2.1%	1.0%	2.6%	1.0%	0.6%	1.2%	0.6%	0.5%
Other	3.5%	3.1%	2.6%	2.5%	2.1%	1.2%	1.8%	1.6%	1.4%
Coryza	2.4%	1.9%	0.9%	2.3%	1.4%	0.7%	1.5%	1.1%	0.7%
Suspect case	72.4%	61.1%	46.3%	67.2%	54.0%	31.2%	66.2%	52.3%	36.8%
Severe case	39.6%	27.5%	18.1%	31.4%	18.0%	11.3%	21.7%	13.4%	10.5%
Number of symptoms	6.9	6.0	4.2	6.6	5.4	3.0	6.2	5.2	3.5
Number of symptoms (IQR)	4	4	7	4	4	6	4	4	6

For all signs and symptoms, among those with a positive test for SARS-CoV-2 proportions were significant different between waves 1 and 2, 2 and 3, and 1 and 3, at 1% with a two-tailed test. Among those with a negative test for SARS-CoV-2, the difference in the proportion for headache was non-significant between waves 2 and 3, and all other differences were significant. For those non-tested, differences in the proportion of odynophagia between waves 1 and 3, rhinorrhea between waves 1 and 2, and shaking chills between waves 2 and 3 were non-significant, and all other differences were significant.

Between the first wave and the third wave, the standardized prevalence of the analyzed signs and symptoms fell, suggesting both a reduction in severity and that presence of signs and symptoms is less sensitive as a method for identifying cases of COVID-19. Among positive COVID-19 cases, the average number of signs and symptoms was 6.9 in the first wave, 6.6 in the second, and 6.1 in the third; among negative cases, the averages were 6.0, 5.4, and 5.2, respectively.

For all signs and symptoms, we rejected the hypothesis of equal proportions between waves 1 and 2, 2 and 3, and 1 and 3 with a significant level of 1% at a two-tailed test for those positive for SARS-CoV-2. For those negative for SARS-CoV-2, only for headache was no difference between waves 2 and 3 (all other differences were significant at 1% for a two-tailed test). For those non-tested, no differences were found for odynophagia between waves 1 and 3, rhinorrhea between waves 1 and 2, and shaking chills between waves 2 and 3.

Using the Ministry of Health's definition of *suspect cases* and *severe cases*, 72.3, and 41.3% met these criteria among those who tested positive in the first wave. These proportions decreased to 66.3 and 18.5% in the third wave. Amongst those who tested negative, these percentages were 60.6 and 28.5% in the first wave, and 52.0 and 12.8% in the third. Among those not tested, 18.8% met the criteria for severe cases in the first wave, decreasing to 9.7% in the third.

**Figure 1:**
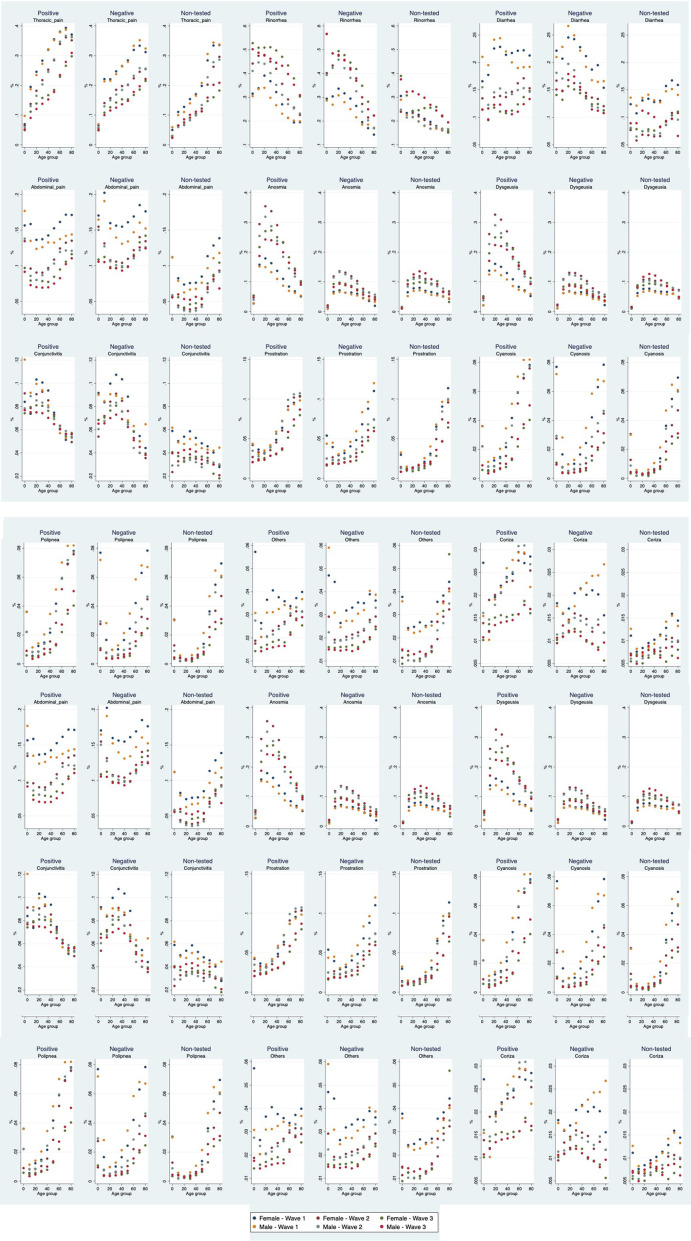
Prevalence of signs and symptoms among those with COVID-like-symptoms that were positive, negative or not-tested for SARS-CoV-2 by wave, age group, and sex.

As presented in Figure 1, there is a clear age pattern in most signs and symptoms for those with a positive test for SARS-CoV-2, except for fever. There are different age patterns: the most common signs and symptoms (cough, headache, myalgia, and arthralgia) present an inverted “U” shape, that is, a higher prevalence among middle-aged groups compared to younger and older ones. For others, such as general debilitation, dyspnea, thoracic pain, and prostration, the prevalence increases with age, with large differences between younger and older groups. Finally, abdominal pain has a “U” shape, with higher prevalence among younger and older groups compared to middle-aged groups. Differences by sex are minor.

For those with a negative test, the patterns are similar, although in this group fever for waves 2 and 3 presents a “U” shaped distribution, that is, higher prevalence among younger and older individuals compared to those middle-aged. Among those not-tested, the most common pattern is increased prevalence with age.

## Discussion

This study analyzes the clinical characteristics of individuals who presented with COVID-like-symptoms at IMSS facilities from March 2020 through October 2021, during which three pandemic waves occurred. Our results show fewer cases were confirmed during the first wave and a higher proportion of those tested were positive compared with the two subsequent waves. In waves 2 and 3, more cases were confirmed, but the proportion tested was higher and the proportion testing positive decreased. These findings were not unexpected, given the increasing availability of both PCR and rapid tests over time.

We also show that the age distribution of patients changed with each pandemic wave, with the younger groups forming a progressively larger share of total patients: those <10 years old went from 1.4 to 4.4%, and similar changes occurred with for those 10 to 19 and 20 to 29 years old. This may be related to susceptibility, as older individuals were affected earlier–and vaccinated earlier– and thus the virus moved to more susceptible individuals.

We also observed significant differences in the presenting clinical characteristics of patients with suspected COVID over time that are consistent with studies in other locations that suggest that viral mutations are related to changes in symptomatology; that is, different viral variants may produce different constellations of symptoms (20), as well as the different severity of disease (21). The proportion of patients presenting with rhinorrhea and odynophagia (runny nose and painful swallowing) increased and the proportion presenting with dyspnea (shortness of breath) and generalized malaise decreased.

When we approached the analysis of the IMSS data on the demographic and presenting clinical characteristics of the population suspected of COVID, we expected to find relatively little change in the demographic characteristics of the population over time and large differences in signs and symptoms due to the differences in severity across variants (20). What we encountered was the unexpected. There were dramatic differences in the age pattern of people becoming infected over time, differences that are explainable in part due to the differential access to vaccines by age. Part of the differences may be due to the fact that the most susceptible populations were infected early and were, therefore, less susceptible in subsequent waves (22), but it is difficult to explain the observed changes with that explanation alone. It may also be that different variants exhibit different age-specific infectivity as has been suggested previously (16, 23). We present no evidence regarding the potential causes of the changes we observed over time but suggest that this is worthy of further epidemiological investigation.

Our analysis identified differences in the clinical profile of untested individuals compared to those tested that are consistent with previous studies in other countries (24), suggesting that at least during the first wave (when the untested represent a larger percentage of all suspected cases), available tests were used preferentially for patients presenting with more COVID-related symptoms. We are unaware of any related explicit policies. However, even in the first wave a preference for testing those who were more symptomatic was not implemented uniformly as 46.6% of the untested cases met the definition of a suspect case and 18.8% of a severe case.

The set of COVID-associated signs and symptoms that have been collected from suspect cases since the beginning of the pandemic do not perform well to predict SARC-CoV-2 infection and have lost specificity over time. This suggests that it would be useful to reevaluate the existing signs and symptoms collected as part of COVID surveillance to question both the utility of continuing to collect the current set as well as the possibility of including others that might improve the predictive value among individuals who seek care.

The main limitation of our study is that data on signs and symptoms are reported by individuals or their relatives, so they may reflect reporting bias. Also, the quality of the data in the reporting system may be variable depending on the data entry process in each facility. Our analysis includes all cases reported by IMSS, the largest provider of health services in Mexico. Also, as the pandemic evolved, greater knowledge of diagnosis and treatment may affect testing decisions. While changes in testing procedures and decisions are not well documented, there is no evidence that this could be related to the reported characteristics of the individuals.

During epidemic surges the common understanding was that any individual presenting with respiratory symptoms was very likely to be a COVID-19 case. However, as the number of cases declines, COVID vaccination rates increase, and isolation measures are relaxed, the probability that a patient presenting with respiratory symptoms has a different infectious etiology increases. It is difficult to imagine that we will ever return to pre-pandemic levels of symptomatic treatment of all but the most severe respiratory infections. It is much easier to imagine that home rapid tests will start to include additional antigens (e.g., influenza virus, RSV, etc.) in addition to SARS-CoV-2 so that surveillance efforts can focus more quickly on outbreaks of atypical pathogens, including new SARS-CoV-2 variants. An alternative future is one where pandemic fatigue dominates and the population tires of distinguishing COVID from other respiratory infections, leading to underestimation of future COVID surges.

## Data availability statement

The data analyzed in this study is subject to the following licenses/restrictions: Access to the administrative data used for this analysis (SINOLAVE) is legally restricted by the IMSS law; authors have been granted access by a research agreement. Sharing of de-identified aggregated data will be considered by authors upon reasonable request. Requests to access these datasets should be directed to jpgutierrez@unam.mx.

## Ethics statement

The studies involving human participants were reviewed and approved by the Research and Ethics Committee (IRB) of the Mexican Social Security Institute (IMSS) (registration number R-2020-785-165). Written informed consent for participation was not required for this study in accordance with the national legislation and the institutional requirements.

## Author contributions

GO and JG conceptualized the study. JG coordinated the analysis. JG and SB drafted the first version of the manuscript. AJ-F and FV participated in the analysis. GO, SB, AJ-F, VB-A, FV, IA-M, and JG reviewed, provided comments, and approved the final manuscript.
